# Darwinian Theory vs. Special Creation

**Published:** 1886-10

**Authors:** 


### DARWINIAN THEORY VS. SPECIAL CREATION.

*Letter from Dr. Park, of Waco.*

EDITOR DANIEL'S TEXAS MEDICAL JOURNAL:

THE educated physician, as a rule, is a close observer of the processes of nature. The laws of growth and development, and all the phenomena of animal life, are inseparably connected with the science of medicine. Some knowledge of chemistry and physics is also absolutely essential to the physician. He finds that the forces and laws governing the development of animal and vegetable life are regular and unvarying, and that nothing is done in a hurried manner, or

*per saltum.* For every effect he feels sure there is an adequate cause, whether he is able to find it or not.

As science advances, the belief in miracles and supernaturalism decreases. The rational mind refuses to accept a supernatural explanation for any phenomenon, when a lower or natural one is at hand. The law of logic, "that we are forbidden to assume the operation of higher causes, when lower ones are found sufficient to explain the observed results," is as binding on us as the multiplication table. This law is the only logical barrier between science and superstition, and, without it, we would lapse into the savage state. The great Kepler violated this law when he accounted for the movements of the planets by saying that each one had an angel behind it, pushing it along in its appointed course. Now, this was excusable in his day, but when Sir Isaac Newton discovered the law of gravitation, which perfectly accounted for all the movements of the heavenly bodies, it became no longer allowable. The rational mind is compelled to accept the lower cause and discard the higher. And so we must do in attempting to explain the phenomena of nature.

There are just two theories offered to account for the origin of all the varied species of animal and vegetable life found upon our globe, and only two. There are none others conceivable. The first, or Mosaic account, is called special creation; the second, or scientific account, is called evolution. The one is miraculous, and comes down to us from an ignorant, barbarous age of the world; the other is derived from the present scientific age. The first has been handed down to us, along with a family of myths, such as a flat earth, solid firmament above, and the rotation of the sun around the earth. All the rest have been gradually dissipated by the light of advancing science, and this one will have to share the same fate. Savage man's interpretation of the phenomena of nature can not stand when opposed to the developments of this cultured age.

The doctrine of evolution and the Darwinian theory are as firmly established, in the minds of scientific men, as the Copernican or gravitation theories. All of them are derived from ligitimate deductions from carefully observed facts; and when this is the case, no scientific theory ever changes.

The opposition to the scientific theory of evolution is confined almost exclusively to the clergy; their opposition is not due to any error which they can point out, but solely because they fear its acceptance will destroy the belief in some of the dogmas of sectarian religion. There are a few others who cling to the old Mosaic account, but in doing so they show to the world that they have not kept up with the science of the day. Twenty years ago there were a few reputable scientific men who disputed the Darwinian theory, but now, as Prof. Marsh truthfully says, "The battle has been fought and won, and it is a waste of time to argue with one who disputes its truth."

To rail at, and attempt to cast ridicule upon, these grand scientific doctrines, is as much out of taste as it would be for a lawyer to deride the learning of Coke and Blackstone in a court of justice.

There can be no objection to any one refusing to endorse evolution or Darwinism until fully satisfied of their truth, but, surely, it is in exceedingly bad taste to rush into print and vehemently abuse them. I would much rather be the author of the Rev. John Jasper's sermon, "The Sun do Move," than to make such a record.

And why not? Since there are some few plausible arguments against the Copernican theory, but none of the slightest force against the theory of evolution.

Mr. Alfred R. Wallace, late President of the British Association, and a man whose fame as a painstaking, clear-headed scientist is not surpassed by any one throughout the whole civilized world; a man noted for his candor and devotion to truth, whose name will live for ages, in a recently published article, uses the following words in reference to Mr. Darwin and his theory:

"However much our knowledge of nature may advance in the future, it will be by following in the pathways Mr. Darwin has made clear for us; and for long ages to come the name of Darwin will stand for the typical example of what the student of nature ought to be, and, if we glance back over the whole domain of science, we shall find none to stand beside him as equals, for in him we find a patient observation and collection of facts, as in Tycho Brahe; the power of using those facts in the determination of laws, as Kepler; combined with the inspirational genius of a Newton, through which he was enabled to grasp fundamental principles, and so apply them as to bring order out of chaos, and illuminate the world of life, as Newton illuminated the material universe. Paraphrasing the eulogistic words of the poet, we may say with a greater approximation to truth,

> "Nature and nature's laws lay hid in night,
> God said, 'Let Darwin be,' and all was light."

Waco, Texas, September 15, 1886.

[While we have objected to the discussion of this subject in Association meetings, as likely to make discord, we see no objection to its discussion in relation to medical science in THE JOURNAL. See disclaimer on first page.—ED.]

